# Understanding caregivers’ and community influencers’ perspectives on the barriers to childhood immunisation in Northern Nigerian States with public-private partnerships in routine immunisation programme

**DOI:** 10.1186/s12889-025-22710-7

**Published:** 2025-04-21

**Authors:** Eno-Obong E. Etim, Angela Odiachi, Leanne Dougherty, Matthew Alabi, Adetayo Adetunji, Adebola Adedimeji

**Affiliations:** 1 Population Council, House 4, No. 16 Mafemi Crescent, Off Solomon Lar Way, Utako District, Abuja, Nigeria; 2 Research Consultant, Abuja, Nigeria; 3 Population Council, New York, USA

**Keywords:** Immunisation, Caregivers, Barriers, Northern Nigeria, Partial adopters, Non-adopters

## Abstract

**Background:**

Despite investments by the Nigerian government and international organisations in childhood immunisation to combat child mortality, coverage in many northern states remains below the national average, thereby increasing the risk of vaccine-preventable diseases. This paper examines the barriers to immunisation in six states in northeast and northwest Nigeria, which have the lowest vaccination coverage rates in the country.

**Method:**

We conducted 24 focus group discussions (FGDs) with mothers/caregivers and community influencers who collaborate with health workers to provide routine immunisation. The socio-ecological framework informed the design of the FGDs. We thematically analysed inductively and deductively coded data on NVivo version 12.

**Results:**

Barriers to immunisation uptake included: Individual and interpersonal level - limited female mobility, adverse events after immunisation; community level - misconceptions and myths about vaccines, religious beliefs and norms, health provider-patient gender discordance, mistrust of immunisation; system level - poor health worker attitude, ineffective documentation of immunisation appointments, distance to health facilities, inadequate human resource capacity at health facilities, vaccine supply shortages, and lack of incentives.

**Conclusion:**

This study highlights the intricate barriers to immunisation uptake in northern Nigeria. Key recommendations include engaging male caregivers through tailored social and behavioural change initiatives and capacity building for health workers to improve their counselling skills on vaccine side effects. Findings from the study provide valuable insights for policymakers and programme implementers. Implementing these interventions can tackle ongoing challenges and improve routine immunisation in these states.

**Supplementary Information:**

The online version contains supplementary material available at 10.1186/s12889-025-22710-7.

## Introduction

Immunisation is among the most effective and cost-efficient public health interventions for reducing child morbidity and mortality [1–3]. For decades, vaccines have safely reduced the burden of diseases like polio, measles, and smallpox, enabling 2–3 million children annually to grow up healthy and thrive. Between 2010 and 2018, the measles vaccine alone prevented 23 million deaths worldwide [4, 5]. However, vaccination coverage has gradually decreased. In 2021, an estimated 25 million children under 1 year did not receive basic vaccines (BCG, OPV, DPT, hepatitis B, measles, yellow fever) globally, with about one-quarter of these children living in Nigeria. This represents six million more children than in 2019 and a decrease in vaccination coverage from 86 to 81% [6, 7]. The COVID-19 pandemic further exacerbated these numbers. A WHO report from 2020 highlighted that global vaccination progress fell short of the targeted 90% coverage, reflecting the serious challenges faced in sustaining immunisation efforts during the pandemic [8]. Additionally, global vaccination trends during this period noticeably declined, with an additional 6 million children reported as unvaccinated or under-vaccinated. The COVID-19 pandemic response was also projected to result in a further decline in vaccination rates in Nigeria [8–10].

The Nigerian government and international organisations have combatted rising child mortality in Nigeria, particularly through interventions aimed at increasing routine immunisation (RI) coverage [11]. Despite these efforts, millions of children remain at risk of death from vaccine-preventable diseases. In 2019, Nigeria topped the World Health Organization’s list of countries with the highest number of under-five deaths [12, 13]. The 2021 Multiple Indicator Cluster Survey (MICS) revealed that 1 in 10 children in Nigeria died before their fifth birthday, and a striking 64% of children aged 12–23 months did not receive all the required vaccines. These rates were much higher in northern Nigeria, where 27% of children were unvaccinated in the northwest and northeast zones, compared to 5–11% in other areas of the country [7].

Immunisation efforts in northern Nigeria face unique challenges due to misconceptions, myths, and false information, resulting in low community demand and vaccine uptake [14, 15]. Thus, the persistence of vaccine-preventable diseases in northern Nigeria has remained a public health concern [16]. Several factors responsible for observed low child immunisation rates include caregivers’ lack of knowledge and/or disapproval, myths about vaccines, fear of side effects, maternal education, socioeconomic status, and a lack of trust in health personnel [15–18]. At the health system level, weak supply chain, poor delivery of services, scarce human resources, funding gaps, poor resource accountability, and weak governance have been cited as potential barriers to uptake [19].

From 2012 to 2022, The Bill and Melinda Gates Foundation (BMGF) and Aliko Dangote Foundation (ADF) partnered with the governments of six northern Nigeria states - Kano, Kaduna, Bauchi, Borno, Sokoto, and Yobe- through a memorandum of understanding (MoU) - to strengthen routine immunisation systems and address funding challenges. The Routine Immunisation (RI) MoU was established primarily to address the inadequate and unpredictable funding for RI in Nigeria, and the suboptimal government commitment to health care, ownership and oversight in northern Nigeria. The programme aimed to enhance partner collaboration to improve vaccine supply chain and logistics, enhance service delivery, address human resource capacity, and increase community trust and demand for routine immunisation in these partner states. The core principles of these partnerships were state ownership, accountability, transparency, and sustainability. To achieve these objectives, the MoUs set up an innovative government-led funding mechanism for RI, encouraging collaboration between government and development partners to improve RI programme performance in programme states. Three pillars undergirded the MoU. First, a shift in donor-government relationships by placing the government at the forefront of RI programme implementation at all levels. Second, conditioning donor support for RI on government co-financing and increasing co-share by the government over four years (30%,50%,70% and 100%, respectively). Third, a focus on strengthening health systems by investing in government staff capacity as a means to promote sustainability rather than setting up parallel donor systems for RI service delivery. This comprehensive approach aimed to create a more effective and sustainable routine immunisation programme in the participating states [20].

To assess these investments, this study, nested in a larger mixed-method evaluation of the RI MoU approach, explored the barriers associated with childhood immunisation uptake in the six MoU states. The purpose is to identify and better understand the persistent challenges that remain despite the enormous investments aimed at improving vaccination coverage. Understanding these barriers will provide useful information for improving RI programmes and informing immunisation policies, especially given Nigeria’s commitment to the Global Vaccine Action Plan (GVAP) [21].

## Materials and methods

### Study area and design

This qualitative study was conducted in the six MoU programme states, namely - Bauchi, Borno, and Yobe (northeast), and Kaduna, Kano, and Sokoto (northwest). Vaccination coverage across these states are low at 5.8% (Sokoto), 13.6% (Borno), 16.5% (Bauchi), 23.0% (Kano), 34.5% (Kaduna), and 49.2% (Yobe) in 2021 [7]. The social-ecological Model (SEM) underpinning this study examines the multiple levels of influence on immunisation behaviour. It posits that behaviour both influences and is influenced by diverse contexts [22]. This model considers interactions between individuals and their social networks, community structures, and institutional/systems/policy contexts. Using this framework, we developed a focus group discussion guide to elicit information from caregivers and community leaders on how these multiple levels constituted barriers to RI uptake.

### Study population

We purposively selected two categories of participants: caregivers and community leaders/influencers. Caregivers were women of reproductive age (15–49 years) who were mothers of children under 2 years who had either partially or not adopted immunisation services. Partial adopters were mothers who had begun but did not complete immunisation for their children, while nonadopters had never accessed immunisation services for their children. Community influencers included traditional, religious, women, and youth leaders, and traditional birth attendants (TBAs), who resided in the study communities and worked alongside the State Primary Healthcare Development Agency (SPHCDA) to implement maternal, neonatal and child health (MNCH) initiatives. To ensure diverse yet representative discussions, we selected two community leaders from each category– using service providers’ records - while maintaining homogeneity in the FGDs. This approach fostered trust, minimised power dynamics, and encouraged participants to share their perspectives freely on the sensitive sociocultural barriers to immunisation in northern Nigeria.

Study participants were recruited with the assistance of health facility workers. These service providers identified potential participants from health facility immunisation defaulter tracking registers and line-listing records of community leaders/influencers associated with the health facilities. The line lists contained key information about all newborns in the communities, collated by service providers and TBAs for community leaders to monitor and track all eligible children for immunisation. Newborns are identified through various methods and at different levels of healthcare, including community birth notifications, health facility birth records, and national population data.

### Data collection

We collected data in 2 local government areas (LGAs) in each study state. While routine immunisation was implemented in all LGAs, we conveniently selected these LGAs based on accessibility and security considerations. Fieldwork was implemented simultaneously in the six states over two weeks between March and April 2022. In each state, a team of three trained and experienced researchers– including two research assistants and one supervisor- worked closely with the service providers and the research staff to ensure that the appropriate participants were recruited. This team consisted of individuals familiar with the subject matter and study region, and fluent in Hausa, the dominant community language. Ultimately, 24 FGDs were conducted, comprising 12 with partial and non-adopters of routine immunisation services and 12 with community leaders/influencers in the study states. We developed all the data collection tools in English and translated them into Hausa using an experienced contracted translator. All FGD guides were pre-tested in a location outside the study communities, and feedback from this testing was used to modify the final guides. The FGDs were conducted face-to-face in Hausa at primary healthcare facilities within each community and lasted about 75 min each. Each interview was facilitated by two bilingual Hausa-English researchers: one interviewer and one notetaker. All study participants provided individual verbal informed consent prior to each FGD. All interviews were audio recorded.

### Data management and analysis

The FGD audio recordings were translated into English and transcribed verbatim by the lead FGD interviewer, following a transcription protocol developed by the research team. Once translated, the data were labelled, de-identified, and securely stored on password-protected computers. The transcripts were then imported into NVivo software (released March 2022) and coded by eight trained coders, some of whom conducted the FGDs. We employed a hybrid deductive-inductive thematic analysis approach by first identifying themes based on the FGD guides and study objectives and subsequently exploring and incorporating emergent patterns and themes in the data into our analytical codebook. For rigour, all eight coders conducted an initial review to familiarise themselves with the data prior to coding. Further, initially, all coders coded the same transcripts to ensure consistency in code application. The resulting discrepancies were then resolved through a consensus-building approach guided by the study objectives. The authors implemented the 32-item Consolidated Criteria for Reporting Qualitative Research (COREQ) checklist in the preparation of this manuscript to ensure comprehensive reporting of qualitative findings [23].

## Results

### Barriers to immunisation uptake

#### Individual and interpersonal level barriers

##### Limited female autonomy and decision-making

Women’s limited decision-making power impacted immunisation uptake. When mothers encountered opposition from social networks such as husbands, parents, in-laws, and other family members, they were unlikely to take their children for immunisation, regardless of their personal views. Such disapproval stemmed from mistrust of vaccines, sociocultural norms, and limited knowledge of the advantages of immunisation.

I was in support of it [immunisation] for my three children at first. But on the third child, his dad [husband] said he was tired of the injection; he did not understand what was going on. You would buy drugs with money, but when you go to the hospital, they give the injection for free. He said we should just stop going for it. However, I did not stop going until I finished collecting five doses for the third child. And for the fourth child, he didn’t support me. He stopped me and said I should not go again.–**Partial-adopter**,** Borno state.**

##### Adverse events after immunisation (AEFI)

Concerns about the side effects of vaccinations also played a role in caregivers’ decision to continue immunisation. Participants reported that their children experienced side effects such as fever and malaise, particularly after receiving pentavalent vaccines. Although some caregivers understood these side effects were transient and typical, many were discouraged and opted not to follow through with the remaining doses. Their husbands also refused them to go for further appointments for the same reasons. Reports of husbands restricting women from taking their children for immunisation were common across the states. This situation was exacerbated by a general mistrust of vaccines. However, in some instances, caregivers did not receive proper counselling regarding these side effects from healthcare providers, contributing to their reluctance to complete the vaccination doses.

The reason why he [husband] stopped me was the children got a fever when we returned [after immunisation]. When he [husband] asked why, and I told him that I collected an injection for them, he asked, “Why?” And that I should not go again. That, on the quest of getting drugs for catarrh, I have brought something new upon the child.–**Partial-adopter**,** Borno state.**

Honestly, I was discouraged by my husband… We recorded a small complication [side effect] in the child, and since then, he [husband] has restricted me from going for another session. He feels the immunisation poisons the child.–**Partial-adopter**,** Kano state.**

#### Community level barriers

##### Misconceptions and Myths about vaccines

Myths regarding conventional medicine were the most cited barrier to immunisation uptake. Caregivers expressed concerns that vaccines could lead to infertility, contribute to the onset of other illnesses, or even result in a child’s death. For instance, one non-adopter in Borno state mentioned that her mother-in-law believed vaccines cause impotency, so she refused to let them take their child to be immunised. Similarly, a non-adopter in Kano state noted, “My in-laws complain that immunisation stops childbirth and sometimes leads to the child’s death.”

##### Religious beliefs and norms

Similarly, barriers stemming from religious beliefs were also widely discussed among caregivers and community influencers. Some community members believed that their religion prohibited vaccination and that seeking care from health facilities altogether was contrary to religious teachings. In Kano and Yobe states, specifically, many community leaders reported that individuals believed that the ability to heal or be disease-free is solely an act of God, attainable only through prayer.

There are those whose religion prevents them from accessing RI. Some of them stay in urban areas, but they reject polio vaccines, and some of them believe that “everything created by a white man is for pagans.” We hear them say these things, and we live with them. Some of them see coming to health facilities as forbidden.– **Male Community Influencer**,** Yobe state.**

##### Health provider-patient gender discordance

Concerns regarding gender discordance between health providers and patients were also raised. In many situations, women often take on the role of primary caregivers for their children, bringing them to health facilities for immunisation. However, when health facility staff were male, some men hesitated to allow their wives to visit the facilities, reflecting deeply entrenched cultural norms about other men having close interactions with their wives, as is often the case in health service provision in the north. One female community influencer from Sokoto noted, “Most of our hospitals don’t have women. According to our tradition in the village, when it’s known that all the staff are male, some people will not allow their wives to go there.” This situation ultimately restricted women and children’s access to necessary health services, including immunisation.

##### Mistrust for community influencers and government/donor intentions

Fears surrounding the motivations of community influencers in facilitating demand also influenced caregivers’ perception of immunisation. Many assumed that these influencers prioritised potential financial compensation from the government for facilitating service use over the community’s health. Additionally, some participants questioned the underlying motivations of governments and donors in providing free vaccines to healthy children, especially when medical care for sick children was not free, raising concerns about a potential hidden agenda. Considering this, many community influencers faced significant challenges in their advocacy efforts. For instance, they frequently encountered hostility when attempting to encourage individuals to get vaccinated. One community influencer recounted an experience.

At times, we encounter numerous challenges. For example, when I try to direct someone to get vaccinated, they often insult me. I once urged a group of people to go and receive the vaccination, but they refused. I persisted and accompanied them to get the vaccine. However, after receiving it, they returned home, and I heard them gossip that they wouldn’t be surprised if I had received money for urging them to get vaccinated. That this is why I advocate for people to get vaccinated.– **Female Community Influencer**,** Borno state.**

Since the day that the announcers came to tell people to go and collect vaccine injections, he [husband] said, “Why would they say that people should go and collect it free of charge since they do not give drugs free of charge when the child is taken to the hospital at the time the child is sick?” They should help with drugs when the child is sick, not when he is healthy.– **Non-adopter**,** Borno state**.

It is noteworthy that this scepticism regarding free vaccines was not limited to caregivers. Surprisingly, some community influencers echoed these sentiments despite being better informed about the importance of routine immunisation and possessing the responsibility to educate and raise awareness among community members.

There was a time when I brought my two children to the health facility, and it happened that free drugs were recently brought to the health facility by the government. They refused to give me [the free drugs], and I complained… I promised not to take my children to a health facility again after that incident. What stops them from giving us free services? Why do they offer free services only when they come to our houses [for immunisation]?– **Female Community Influencer**,** Yobe state.**

#### Systems level barriers

##### Poor health worker attitude

Previous unpleasant experiences with service providers were discussed as a critical barrier to immunisation uptake. Many caregivers reported feeling disrespected by health workers, particularly during previous antenatal and delivery visits. Such absence of respectful care prompted some caregivers to seek care at more distant facilities to avoid future mistreatment. Unfortunately, the high transport costs to these alternate facilities hindered their access to the necessary care.

I ran away from [the facility] because they don’t value their patients; they don’t treat us right in every way. I went there to give birth and was humiliated. Since then, I have discouraged people from going there to access care.– **Non-adopter**,** Sokoto state.**

##### Ineffective Documentation of immunisation appointments

In Borno and Yobe state, some caregivers reported forgetting scheduled immunisation dates because health workers only communicated these appointments verbally, without recording them on appointment cards. When mothers missed appointment dates, they ultimately decided to forego immunisation for fear of possible reprimands from healthcare providers.

They [health workers] honestly don’t write when you should return. They would just tell you to return after four weeks. You could sometimes forget to return.– **Partial-adopter**,** Borno state.**

##### Distance to health facilities

There was a consensus among many participants that the availability and accessibility of health facilities impacted service utilisation. Many emphasised the inadequacy of health facilities in meeting the population’s needs. Community influencers reiterated this concern, highlighting the difficulties in connecting women to routine immunisation services due to the unavailability of functional nearby health facilities, which then imposes a financial burden when individuals have to seek care in distant facilities.

##### Inadequate human resource capacity at health facilities

Insufficient staffing at health facilities resulted in long waiting times for immunisations. Participants indicated that it was common for a single service provider to be solely responsible for multiple tasks, including retrieving vaccines from local government cold stores and administering vaccines– while simultaneously providing other health services and maintaining vaccine records. This overwhelming workload resulted in service delays. Service delays were further compounded by facility personnel prioritising, sometimes inequitably, who received care, causing some caregivers to return home without immunising their children when vaccine supplies eventually became inadequate.

The challenges in our clinic are the workers. The work is too much for one person. Sometimes, when you go, she [service provider] will be doing the immunisation and still writing reports, and sometimes they will look for her at the centre [to undertake other health tasks]. So that is a challenge.– **Partial-adopter**,** Kaduna state.**

Sometimes, you arrive early, but they attend to others who come after you. That was why I stopped going. - **Partial-adopter**,** Sokoto state.**

##### Vaccine supply shortages and stock-outs

Another systems barrier was vaccine stock-outs, particularly in Yobe and Bauchi states. Caregivers reported making multiple visits to health facilities without their children receiving the necessary vaccinations due to stock out. Many mothers shared that returning home with an unvaccinated child after seeking permission from their spouse to visit the facility undermined their credibility and bred suspicion within their households. According to them, their husbands believed that immunisations were being used as an excuse to visit other places.

Some days, you come [visit the facility], and the [vaccine] is finished. Because you told your husband you were going for immunisation, coming back to tell him you didn’t get the immunisation makes him feel like you were lying, and he will not allow you to go back the following week. You see, we are not on our own; we are under him, so we can’t go until he gives us permission. - **Partial-adopter**,** Yobe state.**

*Lack of incentives* was an additional challenge. Community influencers discussed that incentives were vital for encouraging immunisation uptake. Typically, state governments provide mosquito nets to caregivers who bring their children for vaccination. However, some states reported a stock-out of nets for several months preceding data collection. Without incentives, community influencers reported difficulty in persuading mothers to immunise their children.

We used to give out mosquito nets when we administered measles and yellow fever vaccines. Now, it’s been about six months since we ran out of stock, and that is discouraging some of the mothers from coming.– **Female Community Influencer**,** Kaduna state.**

## Discussion

This study qualitatively explored the barriers to routine immunisation across six states in northern Nigeria. The findings suggest that prevalent barriers to immunisation include widespread misinformation and misconceptions about vaccines and socio-cultural and religious norms that foster mistrust in immunisation initiatives. These findings are corroborated by several studies conducted in the region. For instance, recent research conducted in Borno State, northeastern Nigeria, identified rumours and cultural taboos as barriers to vaccine uptake. Similarly, a study from Lagos in southeastern Nigeria highlighted notable scepticism by communities regarding immunisation interventions within the country [24–26]. The nature of these documented suspicions was similar to those identified in this study, as some caregivers expressed the belief that vaccinations were Western clandestine family planning tools that cause infertility and death or acted as mechanisms for population control. Additionally, we found that caregivers, particularly in Kano and Yobe, often struggled with conflicts between their religious beliefs and the utilisation of immunisation services. Many believed that seeking care at a health facility was religiously forbidden, insisting that prayer was the sole means of protection against diseases. Others asserted that orthodox medicine, as well as any intervention from Western sources, are intended only for ‘pagans’. These findings resonate with those from studies conducted in Sokoto and other parts of northern Nigeria, which identified a reliance on faith for disease protection as one of the barriers to vaccine uptake [16, 27]. Importantly, our study highlights the role of community leaders in perpetuating vaccine misconceptions. Some questioned the government’s motives behind providing free vaccines when other essential services for children come with a cost. This raises concerns about the knowledge and credibility of community influencers responsible for promoting immunisation awareness. If these influential leaders hold such views, misinformation regarding vaccination will likely continue to spread within these communities.

We further found that the attitude of health workers was an important factor in the uptake of routine immunisation services, as evidenced by some immunisation studies conducted in Nigeria [24]. Mothers were reportedly reluctant to visit facilities due to previous negative experiences with service providers and fear of being reprimanded for missing immunisation appointments. For instance, one study reported that reprimands from service providers often distressed mothers, leaving them feeling inadequate in their ability to care for their children, which subsequently led to a reluctance to return to health facilities [28]. This highlights the importance of providing respectful care and fostering positive relationships between clients and service providers to enhance vaccine uptake. Poor documentation of vaccine appointment dates was also noted as a systems-level barrier in some states. When these dates were not recorded, many caregivers missed their vaccination appointments. Given that many women may already be navigating the demands of child and home care, immunisation cards serve as a valuable reminder and are a vital health record for tracking immunisation status. These findings are consistent with the 2021 National Immunisation Coverage Survey (NICS), which revealed a direct correlation between low immunisation rates and the lack of available vaccination cards [21]. Furthermore, the failure of health workers to complete immunisation cards raises capacity concerns and may indicate an overworked workforce.

The inability to and delays in accessing health services were also key barriers identified in this study. Many non-adopters cited long distances to a health facility as a barrier to service uptake. Previous research has established that geographic accessibility to health facilities offering routine immunisation services is a crucial determinant of immunisation coverage in northern Nigeria [29–31]. It is, however, worth noting that all focus group discussions for this study were conducted at health facilities within these communities, suggesting that caregivers’ non-prioritisation of immunisation services may be intertwined with distance challenges. More so because we discovered that some caregivers viewed immunisation as unnecessary for healthy children. We further discovered that the lack of effective Adverse Events Following Immunisation (AEFI) counselling and management has perpetuated vaccine-related misconceptions, ultimately contributing to service discontinuation. Caregivers identified vaccine-associated side effects, particularly with pentavalent vaccines, as a major barrier. Many expressed reluctance to return for subsequent vaccine doses following any adverse reactions, especially fever. Such apprehensions may be fuelling community scepticism towards vaccination initiatives. Several studies in northern Nigeria have reported similar findings [27, 32, 33]. For instance, research conducted by Omoleke et al. [34] in Kebbi state, northwest Nigeria, shows that AEFI knowledge among health professionals was suboptimal, with many misinterpreting AEFI merely as vaccine defects or consequences from errors in administration. This underscores the pressing need for targeted interventions aimed at enhancing healthcare providers’ capacity to deliver comprehensive AEFI counselling concerning adverse outcomes following immunisation, alongside a commitment to ongoing capacity building for service providers. Further, this finding may account for the notably low coverage rates of Penta 3 in certain northern Nigerian states, with figures dropping to as low as 11% in regions like Sokoto, a far cry from the Global Vaccine Action Plan target of 90% [21].

Women in Sokoto state also raised discrimination regarding the inequitable prioritisation of who received services, which has been documented as a barrier to immunisation uptake [35]. Additionally, long waiting times emerged as a crucial issue across the states. In this context, delays at facilities were directly linked to staffing shortages. These shortages may have contributed to the poor documentation of vaccine appointment dates and improper counselling on the side effects of immunisation. Furthermore, incentives, such as free mosquito nets, were identified as motivating factors for immunisation uptake; previous studies have also recognised their importance in boosting immunisation uptake [36]. However, despite this evidence, a study conducted in Sokoto state revealed that once incentives are removed, immunisation rates tend to drop back to previous low levels or even lower [15]. Our findings support this claim, as we discovered that some caregivers stopped immunising their children when incentives were no longer provided. This raises sustainability concerns and hints at the potential unintended consequences of using incentives. The demand for incentives appears to be driven by the need for caregivers to feel they receive tangible benefits from immunisation.

Our findings suggest vaccine stockouts in certain states, such as Yobe and Bauchi. During focus group discussions, caregivers reported returning home on different occasions with unvaccinated children due to these shortages. This situation raised suspicion among husbands and created mistrust within households. In a highly patriarchal and conservative region where women’s mobility is often restricted, returning home without an immunised child led husbands to believe that immunisation was being utilised as an excuse for women to visit other places. This poses a challenge to obtaining spousal permission for future immunisation and echoes other studies on the need to engage men through appropriate channels, including traditional institutions [37].

Lastly, conversations with caregivers also noted limited knowledge and awareness of immunisation. Nonetheless, knowledge of immunisation benefits did not always translate to utilising the service. Many mothers, though aware of immunisation benefits, faced opposition from husbands or other influential family members, restricting them from immunising their children. This is similar to other studies conducted in northern Nigeria, where spousal influence was determined as a barrier to immunisation uptake [36]. When confronted with resistance, many women preferred to forgo immunisation services, fearing the potential repercussions for disobedience. A common reason for these refusals was the lack of female health workers at health facilities, which is essential for providing acceptable health services to female clients, particularly given the conservative nature of northern Nigeria.

### Limitations

This study only explores barriers to immunisation from the perspectives of caregivers and community influencers, excluding the views of service providers and male caregivers. As a result, the findings reported here do not comprehensively address system-level (supply-side) challenges, such as vaccine availability, service quality, and workforce issues, which are critical components of immunisation uptake. Furthermore, the exclusion of male caregivers, who often play a central role as decision-makers within households in northern Nigeria, limits the study’s ability to fully capture the gendered dynamics that influence immunisation decisions. Including male caregivers and service providers in future research would provide a more holistic understanding of the barriers to routine immunisation. Despite these limitations, this study contributes significantly to immunisation studies by shedding light on the experiences and perspectives of female caregivers, offering valuable insights for policymakers and programme implementers seeking to improve routine immunisation uptake in northern Nigeria.

## Conclusion

This study highlights the complexity of factors influencing the utilisation of immunisation services in northern Nigeria. Key barriers identified include societal and gender norms, particularly regarding women’s autonomy and decision-making, as well as rumours, poor quality of health services, vaccine mistrust, and poor knowledge of side effects, underscoring the persistent challenges to service uptake. To address these barriers, immunisation programmes should prioritise engaging male caregivers through tailored activities that encourage their support for childhood immunisation. Additionally, targeted training programmes for health workers are essential, focusing on improving their attitudes and communication skills and counselling on vaccine side effects. Regular workshops for health workers could be organised to enhance health workers’ capacity to provide empathetic counselling on managing and addressing concerns about side effects, which many caregivers and their spouses cited as a reason for refusing immunisation. Efforts to dispel myths and misconceptions should leverage social and behavioural change initiatives, ensuring that messages are culturally appropriate and resonate with both caregivers and key decision-makers. Strengthening the capacity of community influencers is also essential, as they are a trusted source of information. Providing them with accurate and contextually relevant messaging will help counter misinformation and foster demand for routine immunisation. These recommendations, if implemented, can help policymakers and programme implementers tackle the barriers to immunisation uptake more effectively, contributing to improved health outcomes for children in northern Nigeria.

## Electronic supplementary material

Below is the link to the electronic supplementary material.

Supplementary Material 1

Supplementary Material 2

Supplementary Material 3

## Data Availability

All data relevant to the publication can be obtained upon reasonable request to the Population Council.

## Abbreviations

ADF: Aliko Dangote Foundation
AEFI: Adverse Events Following Immunisation
BMGF: Bill and Melinda Gates Foundation
FGD: Focus Group Discussion
GVAP: Global Vaccine Action Plan’s goal
LGA: Local Government Areas
MICS: Multiple Indicator Cluster Survey
MNCH: Maternal, Neonatal and Child Health
MOU: Memorandum of Understanding
NICS: National Immunisation Coverage Survey
PHC: Primary Health Care
RI: Routine Immunisation
SEM: Social-Ecological Model
SPHCDA: State Primary Healthcare Development Agency
TBAs: Traditional Birth Attendants
