# Supplementary material for: Understanding caregivers’ and community influencers’ perspectives on the barriers to childhood immunisation in Northern Nigerian States with public-private partnerships in routine immunisation programme
Source: BMC Public Health. 2025 Apr 21;25:1471. doi: 10.1186/s12889-025-22710-7 (PMC12010591; doi:10.1186/s12889-025-22710-7)
Supplement: Supplementary file 1 — Supplementary Material 1 [file 12889_2025_22710_MOESM1_ESM.docx]

**FGD Guide (Non-Beneficiaries- Women/caregivers with children under 2 years of age who have not attended or have attended but NOT fully completed RI program)**

Interviewers note: this is a discussion guide, NOT a questionnaire. Therefore, the focus should be on probing and encouraging the person to talk as much as possible about their experience.

***Interviewer: Hello, my name is _________, and I want to thank you for agreeing to share with me some of your thoughts. We have provided you with informed consent information, and you know what this study is about. Do you have any questions before we begin?***

***Thank you, and welcome to this interview which will be like a conversation back and forth. Your opinions are significant, and no opinion is right or wrong; we want to hear from you.***

| **Participant Demographic Data** |  |
| --- | --- |
| Are you the Caregiver or Mother? |  |
| Mother/Caregiver’s Age: |  |
| Mother/Caregiver’s Gender: |  |
| Highest completed education: |  |
| Age of child/ward |  |
| Gender of child/ward |  |
| Beneficiary or Non-beneficiary |  |
| Other notes: |  |

I have some questions specifically about immunizing your child

1. How many vaccines do you think a child should receive? Why?
2. When do you think a child should be taken for their first vaccination? Why?
3. Where do you think one should go to receive vaccinations? Why?
4. What are the benefits of vaccinations? Explain.
5. What do you think will happen to your child if they do not receive vaccinations?
6. Did your family receive advice regarding when and where to vaccinate your child?
   1. IF YES: Who gave that advice?
   2. IF YES: What did they do to advise or support you? Did you follow their advice?
   3. Did anyone in your family discourage you from vaccinating your child? If so, can you tell me more about what they said and why?
7. What messages have you heard regarding the importance of vaccinating your child? How about other MNCH services?

a. Who did you hear this from? /Where did you hear this from?

Probe: community leaders, health providers

1. Did you take your child to be vaccinated after your child was born?

**If the participant says no, the child was not vaccinated after birth:**

1. Why did you not take your child to be vaccinated?
2. **Follow-up:** Did you find vaccination services were not accessible? Explain.

**Probe**: health worker attitude, service availability, distance to Health Facility, etc

1. **Follow-up:** What concerns do you have about vaccinations?

***Probe:*** knowledge, awareness, personal and traditional beliefs and convictions, efficacy, safety etc.

**If the participant says the child was not fully vaccinated after birth:**

1. Why was your child not fully vaccinated?

Probe: Poor service provision, socio-economic status, poor knowledge and perception, distance to HF, the attitude of healthcare workers, poor documentation of vaccination visits, etc.

1. **Follow-up:** What concerns do you have about vaccinations?

***Probe:*** knowledge, awareness, personal and traditional beliefs and convictions, efficacy, safety, distance etc. Thank you for your time.
