# Supplementary material for: Understanding caregivers’ and community influencers’ perspectives on the barriers to childhood immunisation in Northern Nigerian States with public-private partnerships in routine immunisation programme
Source: BMC Public Health. 2025 Apr 21;25:1471. doi: 10.1186/s12889-025-22710-7 (PMC12010591; doi:10.1186/s12889-025-22710-7)
Supplement: Supplementary file 2 — Supplementary Material 2 [file 12889_2025_22710_MOESM2_ESM.docx]

**FGD Guide: Community Structures (VDCs, WDCs, community volunteers, TBAs, Religious Leaders, Traditional leaders)**

Interviewers note: this is a discussion guide, NOT a questionnaire. Therefore, the focus should be on probing and encouraging the person to talk as much as possible about their experience.

***Interviewer: Hello, my name is _________, and I want to thank you for agreeing to share with me some of your thoughts. We have provided you with informed consent information, and you know what this study is about. Do you have any questions before we begin?***

***Thank you, and welcome to this discussion which will be like a conversation back and forth. Your opinions are significant, and no opinion is right or wrong; we want to hear from you.***

| **Participant Demographic Data** |  |
| --- | --- |
| Age: |  |
| Gender: |  |
| Title/role in community structure |  |
| The number of months/years in the position: |  |
| Highest completed education: |  |
| Other notes: |  |

**A. Introduction to the discussion**

***[Make sure all these questions are asked]***

For starters, I'd like to know more about you.

1. Please tell me a bit about your role in the community.

**B. Community Linkages**

**[Interviewer]** Great, thank you for sharing this with me. I want to ask you a few questions to learn more about your experience with activities to generate community engagement with the health facility.

1. Describe the community/traditional structure for RI engagements
2. What community engagement strategy is in place to ensure community participation in the planning and implementation of RI interventions?
   1. **Probe** for the involvement of VDCs, TBAs, WDCs, Traditional Council and Religious leaders
3. To what extent do you feel the community structures are involved in microplanning and review of RI activities in RI MOU states and RI/PHC activities in RI/PHC MOU states- fixed or outreach sessions? What are their responsibilities?
   1. **Probe** for community insight, area mapping, awareness creation, mobilization of children under-2 etc.

4a. How do community structures create demand for RI or vaccination in the community?

- 1. Probe for the platforms used by the VDCs, WDCS, Traditional and Religious leaders.
  2. Probe for community referrals and defaulters tracking

4b. How do community structures create demand for other PHC services (Probe for platforms for other MNCH services -ANC, FP, Nutrition, etc.)

1. What are the barriers to accessing RI and MNCH services (ANC, FP, Nutrition) identified in the communities?
   1. **Probe:** Social and religious norms
   2. **Probe:** Availability/access/distance to the vaccine and waiting time at HF
   3. **Probe:** Attitude of healthcare workers
   4. How do community structures work to address these barriers?
2. What challenges do you experience in defaulters tracking and mobilizing eligible children for immunization and women for ANC services?
   1. **Probe** for vaccine hesitancy and why.
   2. **Probe** for lack of microplanning, communication, mobilization or data compiling and reconciliation skills.
   3. **Probe** for challenges relating to motivation and working conditions for community actors
   4. **Follow-up:** Have these challenges been addressed? If yes, how were you able to address these challenges? If No, why not? How can these challenges be addressed?
3. Has the caregiver's attitude towards health-seeking or RMNCH services uptake changed? Share examples with stories.
   1. **Probe:** Is there a change in knowledge, attitude, and behaviour towards RMNCH?
   2. **Follow-up**: Has demand for RI increased in the HFs? Is the demand sustained over a period?
4. Overall, what lessons have you learned from supporting RMNCH in your state?
   1. **Follow-up**: If you were going to change some things in the way the RI/PHC system is coordinated or organized, what would they be and why?
   2. **Follow-up**: In your opinion, which aspects/components of the RI/PHC system are working effectively, and which do you think need improvement?
   3. **Follow-up**: What is your recommendation for the government, donors and health providers to improve the intervention?
5. What else would you like to discuss on RI/PHC MOU that we have not discussed?
