# Supplementary material for: Understanding caregivers’ and community influencers’ perspectives on the barriers to childhood immunisation in Northern Nigerian States with public-private partnerships in routine immunisation programme
Source: BMC Public Health. 2025 Apr 21;25:1471. doi: 10.1186/s12889-025-22710-7 (PMC12010591; doi:10.1186/s12889-025-22710-7)
Supplement: Supplementary file 3 — Supplementary Material 3 [file 12889_2025_22710_MOESM3_ESM.docx]

**FGD Guide (Beneficiaries - Women/caregivers with children under 2 years of age accessing RI and other PHC programs)**

Interviewers note: this is a discussion guide, NOT a questionnaire. Therefore, the focus should be on probing and encouraging the person to talk as much as possible about their experience.

***Interviewer: Hello, my name is _________, and I want to thank you for agreeing to share with me some of your thoughts. We have provided you with informed consent information, and you know what this study is about. Do you have any questions before we begin?***

***Thank you, and welcome to this interview which will be like a conversation back and forth. Your opinions are significant, and no opinion is right or wrong; we want to hear from you.***

| **Participant Demographic Data** |  |
| --- | --- |
| Are you the Caregiver or Mother? |  |
| Mother/Caregiver’s Age: |  |
| Mother/Caregiver’s Gender: |  |
| Highest completed education: |  |
| Age of child/ward |  |
| Gender of child/ward |  |
| Beneficiary or Non-beneficiary |  |
| Other notes: |  |

I have some questions specifically about immunizing your child

1. How many vaccines do you think a child should receive? Why?
2. When do you think a child should be taken for their first vaccination? Why?
3. Where do you think one should go to receive vaccinations? Why?
4. What are the benefits of vaccinations? Explain.
5. What do you think will happen to your child if they do not receive vaccinations?
6. Did your family provide advice regarding when and where to vaccinate your child?
   1. IF YES: Who gave that advice?
   2. IF YES: What did they do to advise or support you? Did you follow their advice?
   3. Did anyone in your family discourage you from vaccinating your child? If so, can you tell me more about what they said and why?
7. Did you take your child to be vaccinated after your child was born?

**If the participant says yes, the child was vaccinated after birth:**

1. How long after your child was born did you get your child vaccinated?
2. Why did you take your child to be vaccinated?
3. Tell me about your experience.
4. Which vaccine did your child receive?
5. Were vaccines available?
6. Was a health provider available?
7. Was your child vaccinated?
8. Did the health provider encourage you to vaccinate your child?
9. Did the health provider suggest you return for another vaccination? If yes, did the health provider provide you with a date to return?
10. What other services have you accessed at the facility asides from vaccination within the last 6 months?

Probe for: services under the PHC Minimum Service Package (particularly MNCH services such as ANC, Family planning, Child Health, Nutrition, Malaria, etc.)

1. What can you say about the quality of service you received?

**Probe:** for waiting time, discussion on the type of vaccine and side effects; nutrition – exclusive breastfeeding, health worker attitude, pricing, and costs of services and commodities)

- 1. Would you say you were satisfied with the service rendered? Why/why not? Provide reasons for your answer
  2. What can you say about the attitude of health care providers? Please describe how they make it easier or challenging to access services

1. What messages have you heard regarding the importance of vaccinating your child? How about other MNCH services such as ANC, FP, Nutrition, Child Health)

a. Who did you hear this from?/Where did you hear this from?

Probe: community leaders, health providers

1. If you were going to change how the vaccination and other MNCH services are offered, what would they be and why? Thank you for your time.
